# Heterogeneous learning cultures in interprofessional education: a teacher training

**DOI:** 10.3205/zma001232

**Published:** 2019-05-16

**Authors:** Ronja Behrend, Mira Mette, Maud Partecke, Kathrin Reichel, Birgit Wershofen

**Affiliations:** 1 Charité - Universitätsmedizin Berlin, Dieter Scheffner Fachzentrum für medizinische Hochschullehre und Ausbildungsforschung, Prodekanat für Studium und Lehre, Berlin, Germany; 2 Medical Faculty Mannheim, Heidelberg University, Division for Study and Teaching Development, Mannheim, Germany; 3 Universitätsmedizin Greifswald, Klinik für Anästhesiologie, Anästhesie-, Intensiv-, Notfall- und Schmerzmedizin, Greifswald, Germany; 4 Federal Institute for Occupational Safety and Health, Division “Work and Health”, Berlin, Germany; 5 University Hospital, LMU Munich, Institute for Medical Education, Munich, Germany

**Keywords:** Interprofessional learning, interprofessional education, teaching and learning cultures, heterogeneity, health professions, teacher training

## Abstract

**Aim: **Due to the political demand for the integration of interprofessional (IP) learning into the undergraduate education of health professionals, teachers now have to create and perform IP courses. The IP and thus heterogeneous learning groups pose a special challenge. The presented project aimed at designing a workshop training to support teachers to reflect on heterogeneous learning cultures and to prepare for IP teaching.

**Methods: **The workshop concept was developed in using the Plan-Do-Check-Act (PDCA) cycle and included planning, several rounds of testing and the evaluation of the concept. All planning steps in the development of the workshop concept followed the principles of cooperative learning. The concept evolved in an iterative process based on participants’ feedback and facilitators’ self-reflection.

**Results: **The resulting workshop concept includes theoretical input as well as discussion, teamwork and participants’ self-reflection. The workshop’s core element is the work assignment to develop an IP teaching session considering different learning cultures. Work results and experiences are discussed with the entire group and required skills of IP teachers are identified.

**Conclusion: **The subjective feedback of participants regarding their satisfaction and knowledge gained indicates that the workshop concept is well received. The joint planning of an IP teaching session highlights particularities resulting from heterogeneous learning cultures. These should be utilized in IP education to better prepare learners for IP cooperation in the workplace.

## Zusammenfassung

**Zielsetzung:** Aufgrund der politischen Forderung nach Integration von interprofessionellem (IP) Lernen in die Ausbildungen der Gesundheitsberufe stehen Lehrende vor der Herausforderung, IP Lehrveranstaltungen zu konzipieren und durchzuführen. Die IP und damit heterogenen Lerngruppen stellen dabei eine Besonderheit dar. Ziel des vorgestellten Projekts war die Entwicklung eines Schulungskonzeptes in Form eines Workshops, in dem Lehrende über heterogene Lernkulturen reflektieren und auf die IP Lehre vorbereitet werden.

**Methodik: **Die Entwicklung des Workshopkonzeptes erfolgte entsprechend des „Plan-Do-Check-Act-Zyklus“ (PDCA-Zyklus) und umfasste die Planung, die mehrfache Erprobung sowie die Evaluation des Konzeptes. Alle Planungsschritte bei der Entwicklung des Workshopkonzeptes erfolgten nach den Prinzipien des Kooperativen Lernens. Das Konzept wurde iterativ auf Grundlage von Teilnehmerfeedback und Selbstreflexion der Referentinnen stetig weiterentwickelt.

**Ergebnisse:** Das entwickelte Workshopkonzept beinhaltet Theorieinput sowie Austausch, Zusammenarbeit und Reflexion der Teilnehmenden. Kernelement des Workshops ist der Arbeitsauftrag, eine IP Lehrveranstaltung zu entwickeln und dabei lernkulturelle Besonderheiten zu berücksichtigen. Arbeitsergebnisse und -erfahrungen werden im Plenum diskutiert und notwendige Fähigkeiten von IP Lehrenden identifiziert.

**Schlussfolgerung: **Die subjektiven Rückmeldungen der Teilnehmenden zur Zufriedenheit und zum Erkenntnisgewinn lassen auf eine Akzeptanz des Workshopkonzeptes schließen. Durch die gemeinsame Planung einer IP Lehrveranstaltung werden Besonderheiten verdeutlicht, die sich aus heterogenen Lernkulturen ergeben. Diese Besonderheiten gilt es beim IP Lehren zu nutzen, um die Lernenden besser auf die IP Zusammenarbeit in der Praxis vorbereiten zu können.

## 1. Introduction

In the light of increasingly complex health care needs, cooperative working approaches are becoming more and more important. National and international health care and education experts recommend that health professionals (e.g. in medicine, nursing, physiotherapy and occupational therapy) need to be prepared for interprofessional (IP) cooperation already in their undergraduate education and that shared learning opportunities should be implemented in existing curricula [1], [2], [3], [4], [5]. IP learning takes place when two or more professions learn with, from and about each other to improve collaboration and the quality of care [6]. The skills required for this are summarized in various international IP competency frameworks [e.g. [7], [8], [9]]. Even though there is no German-language framework for IP competencies yet, the need for acquiring IP competencies has also been recognized by German health professionals [5], [10]. 

In 2012, the Robert Bosch Stiftung initiated the program “Operation Team – Interprofessional Learning in the Health Professions” to implement IP learning in a targeted manner at university medical departments throughout Germany [11]. However, the implementation proved to be challenging because health professionals in Germany are trained in different qualification paths, which entail different teaching and learning behaviors [12], [13], [14]. This is why, in the course of the program, an increasing need was identified to inform interested teachers and curriculum developers about how to develop concepts and implement IP teaching and learning offers. 

### Heterogeneous learning cultures

Heterogeneity in groups of learners is a challenge in any educational setting. In the context of IP education, this aspect takes on a specific meaning as it refers to the composition of groups of learners being trained in different professions. In this context, the focus is less on vertical heterogeneity (i.e. differences in performance levels) and more on horizontal heterogeneity in terms of approaches and qualitative characteristics [15]. The different conditions for education in the health professions, i.e. academic or non-academic, entail different concepts of educational theory and learning methods that affect learners’ educational experience and socialization processes and shape their learning expectations and habits. Through training and work in a certain profession, occupation-specific, shared attitudes, knowledge, norms, values and behaviors are learned or acquired [16] that contribute to the creation of professional cultures and manifesting specific learning cultures [13], [17], [18], [19]. Learning cultures are shaped by the learners and teachers themselves, by the characteristics of the educational institution (school, university, curricula, teaching conditions, etc.), pedagogical interventions and didactic design and also by the self-image of the individual professions [17], [18]. Especially educators shape learners, as they act as role models and therefore impact on the learning experience [17]. The cultures developed in the monoprofessional training paths lead to heterogeneous learning and heterogeneous professional cultures. Health professions hold specific characteristics that show up as heterogeneity factors in IP learning groups, such as different levels of knowledge, reference disciplines, problem-solving approaches, terminologies and tasks in patient care. 

Teachers often regard heterogeneous classes as making their job more difficult [20]. As the aim of IP education is, that participants with different learning cultures learn in groups with, from and about each other, it is necessary to specifically include these aspects in the didactic planning and train teachers to develop and implement these teaching formats. The qualification of teachers in the health professions in general has been proven to be important [21], [22], [23] and is increasingly taking priority in the field of IP education as well [24]. The need for qualification also results from the health professions’ lack of knowledge about each other, because not all curricula include sharing knowledge about other health professions. Since IP teachers themselves were trained monoprofessionally, the qualification for IP education is particularly relevant. 

To our knowledge, up to 2016, there was no training program in Germany that addressed heterogeneous learning cultures in IP education of health professions. 

The following project description therefore describes the planning, implementation and further development of a workshop concept that prepares teachers of different health professions for the task of designing and implementing IP lessons. As result, the workshop concept is presented in detail, which focuses on dealing with the heterogeneity of learning cultures. 

## 2. Project description

### Development of the workshop concept

**Figure 1:**
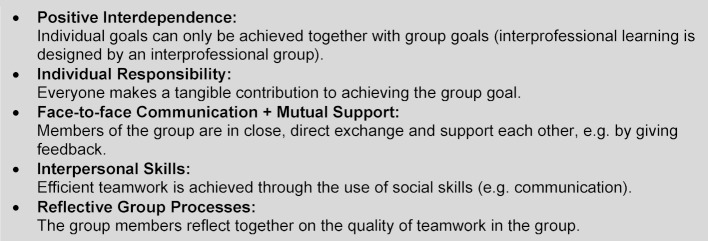
Cooperative learning – design principles [26]

The development of the workshop concept was initiated by an IP working group consisting of five project coordinators from different professions (physiotherapy, occupational therapy, nursing science, education and the humanities), who have been entrusted with developing and implementing IP teaching and learning projects at various universities since 2013. The development process followed the PDCA (Plan, Do, Check, Act) cycle [25], which allows a structured approach. In the planning phase (“Plan”), the IP working group met and defined the objectives, structure and content of the first workshop. The content, such as the theoretical input or the work assignment for the workshop participants, was developed in teams of two and then discussed and determined by the whole group. In didactic terms, the design of the workshop was largely based on the principles of cooperative learning (see figure 1 (Fig. 1)). Cooperative learning was chosen because this method gives all group members equal rights, encourages them to support each other and lets each individual contribute to achieving the common learning goals or results [26], [27].

The workshop was held at three different German-language congresses between April 2016 and March 2017 (“Do”). At least two members of the working group conducted each workshop as facilitators. Each time, the workshop title and duration were adapted to the framework set by the congress organizers. Between eleven and 25 people from at least three different professions took part in each of the workshops, most of them teachers for health professions, such as medicine, nursing, physiotherapy, occupational therapy or speech therapy. At the end of the workshops, participants reflected in an open feedback session on three key questions:

- What is your take-home-message from the workshop?
- Were your expectations met?
- What other feedback would you like to give to the facilitators?

In addition, the responsible workshop facilitators reflected in writing on improving the workshop with regard to the structure, the methods and the content of the workshop (“Check”) [28]. The explorative feedback from a total of 79 participants and the reflections of five facilitators were integrated in a formative way into the further development of methods and content of the workshop concept [29] (“Act”). Table 1 (Tab. 1) provides information on the findings of the evaluation for each of the three workshops. 

Based on the above described feedback and reflections, participants were given more opportunities to become actively involved from the second workshop onward. The work assignment was elaborated in more detail and a handout was developed to facilitate the task. In addition, more time was given for the reflection of the different teaching and learning cultures in order to enable a more in-depth exchange between the participants.

No personal data were stored or processed for the oral participant feedback. There was no negative consequence for workshop participants who decided not to participate in the feedback round.

## 3. Results

### Workshop concept

The result of the planning, implementation and evaluation process described above (see table 1 (Tab. 1)) is a workshop for IP teachers and curriculum developers in therapeutic and diagnostic professions, nursing and medicine. Participation in the workshop does not require any previous knowledge, but basic knowledge of the different health professions is of advantage. The goal is an IP composition of the group of participants from the different health professions mentioned above. The maximum group size has been set at 20-25 participants. The three-hour workshop was held for the first time at the annual conference of the Gesellschaft für Medizinische Ausbildung (GMA) 2017 in Münster, Germany, titled “Heterogeneity of learning cultures in the health professions – A challenge for interprofessional teaching and learning”; the workshop had 20 participants (see table 2 (Tab. 2)).

#### Learning objectives

The learning objectives for the workshop participants are: The workshop participants…

- reflect on their own learning and teaching culture;
- get to know learning and teaching cultures of other health professions;
- develop a didactic concept of an IP teaching session considering heterogeneous learning cultures;
- identify specific requirements for their own teaching activities in the context of heterogeneous learning cultures in IP teaching settings.

#### Workshop agenda

##### 1. Introduction

Besides the usual introduction to workshops (see table 2), the heterogeneity within the group of workshop participants is demonstrated by asking people for their professions. Leading to the main topic of teaching and learning cultures, terms about certain characteristics and preferred learning styles are presented and assigned to specific health professions by the participants. 

##### 2. Input

The introductory presentations at the beginning of the workshop were aimed at creating a shared knowledge base among the participants. They included a definition of professional cultures: “Culture always manifests itself in an orientation system typical for a nation, society, organization or group. This orientation system is made up of specific symbols (e.g. language, gestures, facial expressions, clothing, welcoming rituals)” [30].

In the next step, examples from the professional context illustrate how members of a particular profession show specific symbols, e.g. in language or clothing. Participants are asked to think about how they experienced their own learning and themselves as a learner in their own undergraduate education. For this purpose, group members are teamed up in small groups to exchange their thoughts. The idea is to show that learning is different from person to person and was experienced differently by the participants in their respective undergraduate education (e.g. practical training with patients as opposed to fact-based textbook lectures).

Next, based on their reflections, participants should recognize that their own learning experience also influences their teaching. A presentation follows that defines IP learning, presents some IP competency frameworks and gives recommendations for implementing IP learning objectives. Particular attention is paid to the key role of the teachers.

##### 3. Work assignment

**Figure 2:**
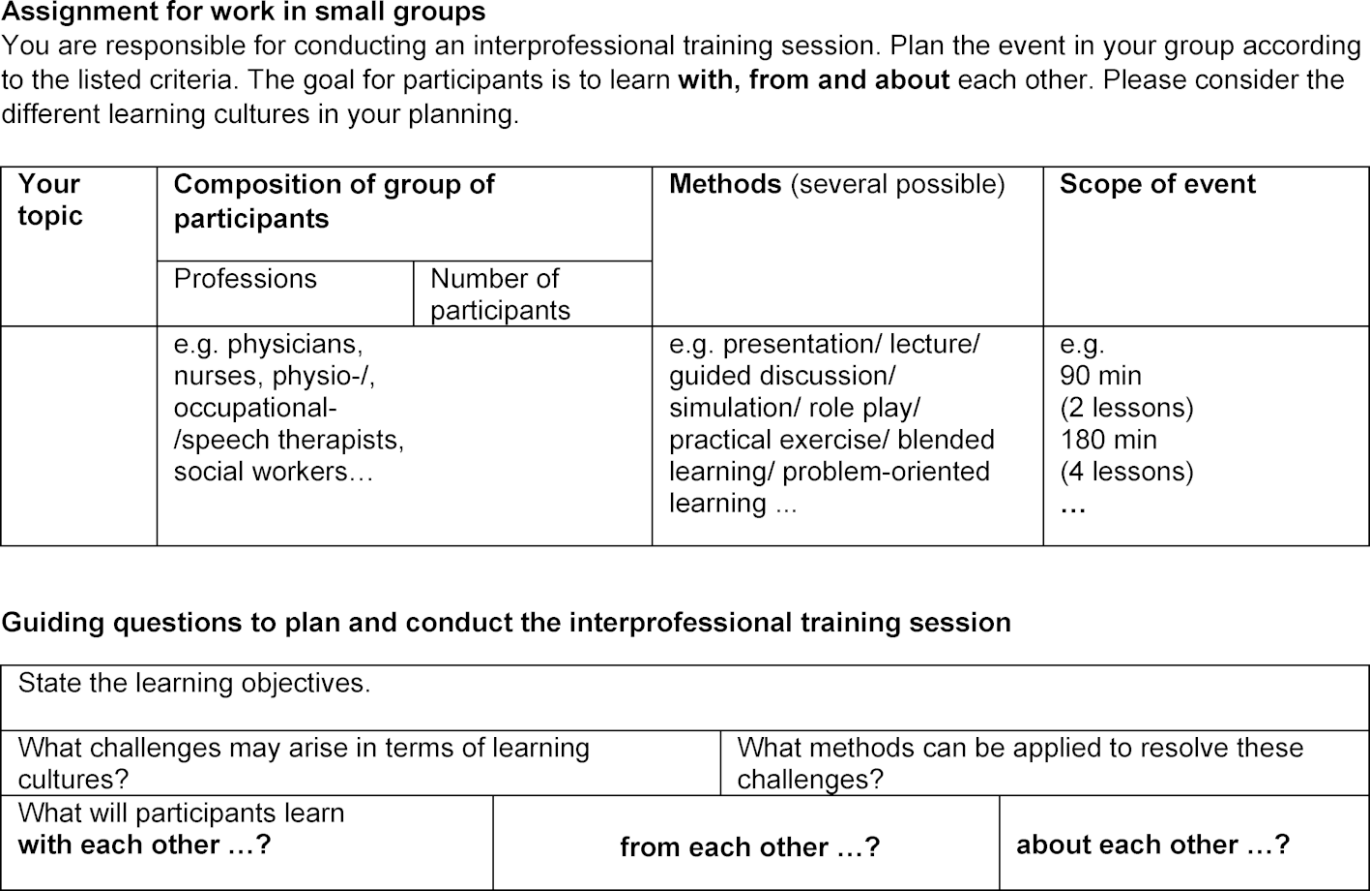
Handout for group work

This part of the workshop builds on active teamwork in small IP groups. The goal of each group is to develop an IP course. The aim is not only to implement the methods for learning with, from and about each other, but also to explicitly consider and address differences in learning cultures. To create a positive interdependence according to the characteristics of cooperative learning, the members of the small group determine the topic of the lesson themselves. Each group member is asked to contribute their professional perspective to the planning process and exchange ideas with the others. The IP group composition offers the opportunity to deal with different teaching and learning cultures during the planning process and to personally experience IP cooperation. A handout with characteristic features of IP educational formats supports the teamwork assignment (see figure 2 (Fig. 2)). 

##### 4. Exchange with the entire group and reflection

The results of the work assignment are presented to the entire group. In a reflective process, a design principle of cooperative learning, participants reflect on how they experienced the teamwork in their small IP groups, how challenges were resolved constructively and what influence the different professional cultural backgrounds had.

Based on the experience gained in the group work, participants define the skills of teachers that ensure the success of IP education. This is followed by a discussion in which the participants can ask the facilitators questions and share their experiences. 

## 4. Discussion

The participants’ feedback indicates that the workshop with its chosen content and didactic methods is suitable for reflecting on the different learning cultures and applying the findings to designing an IP course. According to the workshop facilitators, the theoretical input at the beginning of the workshops proved to be useful, as it created a shared knowledge base for all participants. This was confirmed by their feedback. The methods chosen for the interactive workshop allowed an in-depth exchange among participants, which led to lively discussions in the workshops. Particularly during the small group assignment, intensive discussions took place. The subsequent reflection on the teamwork with the entire group showed that a learning process was triggered by the activity.

The participants’ feedback was positive from the beginning with regard to the workshop agenda and content. The measures for improvement related to the examination of the teaching and learning cultures, which were increasingly given more time in terms of content and discussion. The first workshop was attended by eleven participants and all subsequent workshops were fully booked, which can be interpreted as a positive indication of the increasing interest in the topic.

As described in the introduction, heterogeneity is frequently considered a burden, however, it can also be an opportunity for learning [20]. A benefit of IP learning may be that working together in heterogeneous learning groups prepares learners better for IP patient care in their subsequent workplace. The discussion among the participants and with the facilitators stressed specific requirements for IP teachers. In addition to general teaching skills, such as expertise, methods competence or communication skills, the participants indicate some more skills as important for IP teaching, such as a person’s attitude, the ability to teach in an IP team and the non-judging or value-enhancing presentation of different professional perspectives. 

The unpredictable expectations, previous knowledge, group size and professional backgrounds of the workshop participants were a challenge in conducting the workshops. These brought different dynamics to each of the workshops and had an impact on the focus of the respective workshops, which required flexibility on the part of the facilitators. 

A strength of the workshop concept is that participants with different levels of knowledge and different perspectives can, and are encouraged to, join. Due to the structured approach in planning and the several rounds of testing, different IP perspectives were integrated into the presented workshop concept. Teams of IP teachers could be helpful to take up the different perspectives. The workshop also offers room for a general discussion of IP teaching and learning, as there is no pressure of direct implementation. Conducting the workshop as part of conferences offers the possibility of benefiting from the experiences of different institutions. A limiting factor is the individual conditions at each university, which play a major role in developing and implementing IP education. The implementation at a specific location and of specific formats must always be adapted to the local conditions, so that the workshop can supplement the training for teachers without replacing the specific preparation for IP teaching at the individual locations. 

## 5. Conclusion

To allow IP education to succeed, different learning cultures should be considered in the methods and didactic approaches. These manifest themselves, for example, in different learning habits or certain didactic methods for which teachers should be specifically prepared. The workshop concept presented in this article can serve as a model for IP teachers and university departments to raise awareness among teachers for heterogeneous learning cultures. This can be useful to complement the university’s didactic preparation for specific formats to recognize the needs of learners and address them in teaching. In further evaluations of future workshops, it could be examined to what extent the participants actually show improved IP teaching skills after the workshop. 

## Funding

The workshops were funded by the Robert Bosch Stiftung.

## Competing interests

The authors declare that they have no competing interests. 

## Figures and Tables

**Table 1:**
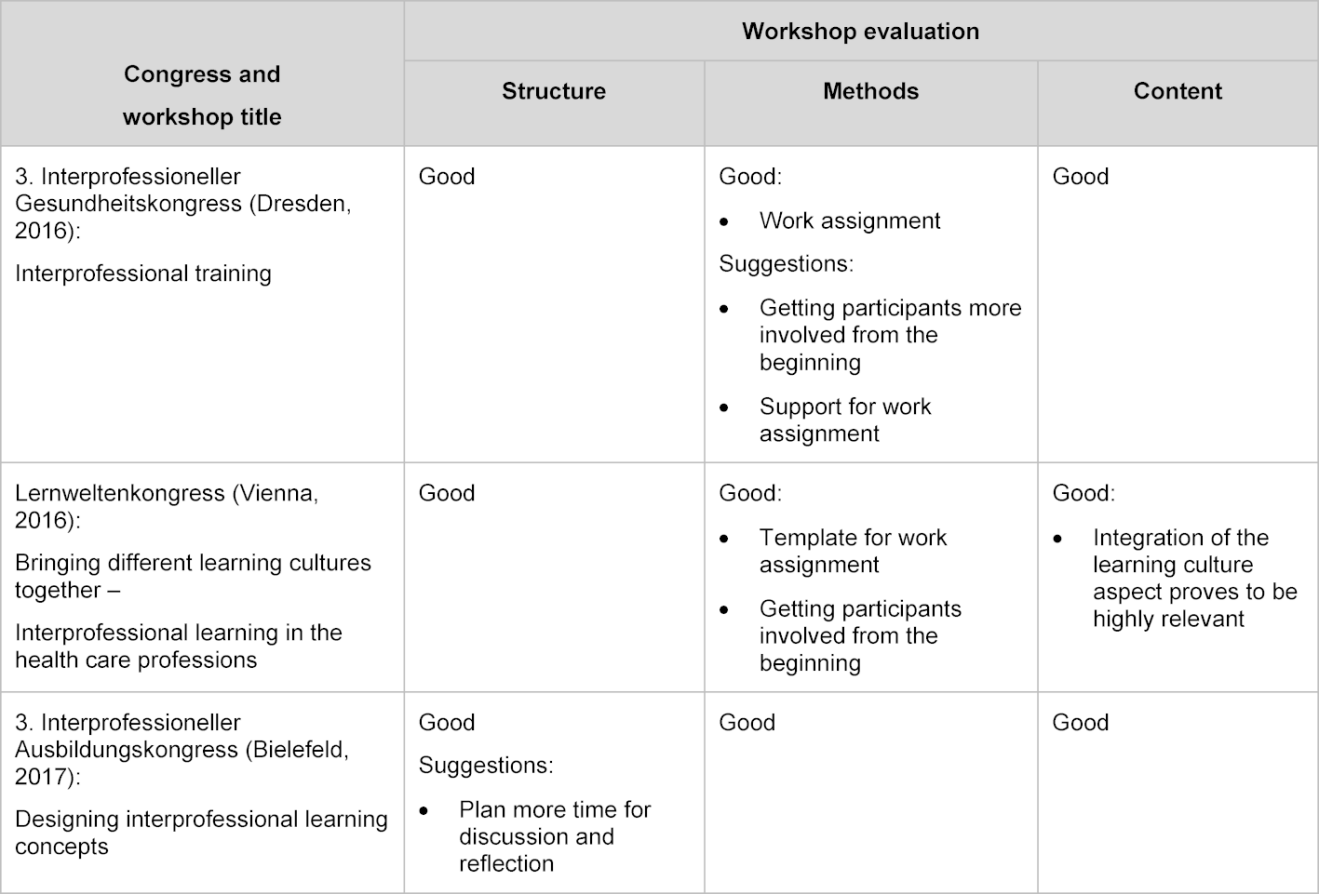
Overview of conducted workshops and evaluation

**Table 2:**
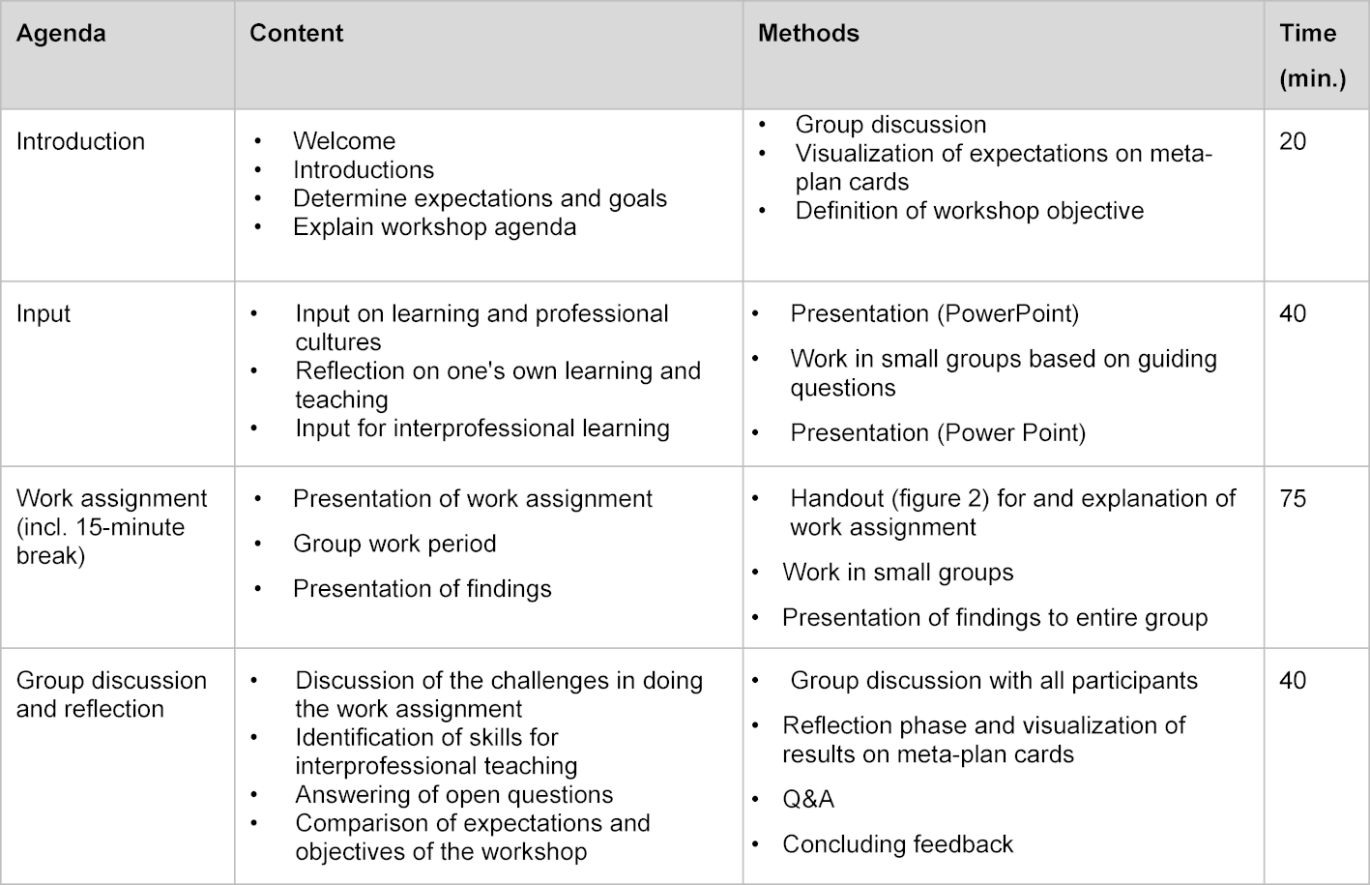
Agenda, content and methods of the workshop concept
